# Robotic Ivor-Lewis Esophagectomy for Corrosive-Induced Esophageal Stricture

**DOI:** 10.7759/cureus.23738

**Published:** 2022-04-01

**Authors:** Vaibhav K Varshney, Raghav Nayar, Selvakumar Balakrishnan, Chhagan L Birda

**Affiliations:** 1 Surgical Gastroenterology, All India Institute of Medical Sciences, Jodhpur, Jodhpur, IND; 2 Gastroenterology, All India Institute of Medical Sciences, Jodhpur, Jodhpur, IND

**Keywords:** benign esophageal stricture, intrathoracic anastomosis, corrosive esophageal stricture, robotic ivor-lewis, minimally invasive esophagectomy (mie)

## Abstract

Corrosive-induced stricture of the esophagus is associated with long-standing morbidity. Though required in particular situations, esophagectomy circumvents the long-term complications of the remnant scarred native esophagus. We performed a robotic Ivor-Lewis esophagectomy for corrosive esophageal stricture and demonstrated its feasibility for the same. A young male patient presented with a history of caustic ingestion, leading to a long segment stricture in the lower third of the esophagus. He developed absolute dysphagia, which was refractory to endoscopic dilatation. A robotic approach was utilized to create a gastric conduit followed by intrathoracic esophagogastric anastomosis. He had a smooth postprocedure course, was discharged on a soft diet on the seventh postoperative day, and is doing well after six months of follow-up. The robotic Ivor-Lewis approach can be safely performed for corrosive esophageal stricture, akin to esophageal malignancy. Besides the comfort of performing the procedure, especially intra-thoracic anastomosis, it helps alleviate the chances of mucocele formation and sequelae of cervical neck anastomosis.

## Introduction

Corrosive-induced esophageal stricture (CIES) demands surgical intervention when it is either refractory or not amenable to endoscopic treatment. The diseased esophagus is frequently bypassed and replaced by the stomach, colon, or jejunum, depending on the location and extent of the injury. The resection of the scarred esophagus is fraught with difficulties such as dissection of the scarred peri-esophageal tissue, damage to adjacent important mediastinal structures, and risk of bleeding [1]. On the other hand, there is an increased chance of mucocele formation and malignant transformation in the innate esophagus after the bypass procedure [2]. Open techniques have proven to be highly morbid in these nutritionally debilitated and fragile patients. A minimally invasive esophagectomy can evade both the procedure-related short-term problems and long-term consequences related to the remnant esophagus [3,4].

We present a case of CIES in the lower esophagus that was managed by robotic Ivor-Lewis esophagectomy (RILE) with intrathoracic esophagogastric anastomosis. This approach has not been described previously in the literature in the context of esophageal stricture secondary to corrosive ingestion. We highlight the feasibility, ease, and safety of performing this procedure for CIES.

## Case presentation

A 26-year-old male presented to the outpatient department with a history of acid ingestion accidentally nine months back. On initial evaluation, his upper gastrointestinal (UGI) endoscopy revealed grade III esophageal injury (Zargar Classification), which was managed conservatively. He developed progressive dysphagia over the next three months and could not swallow saliva. A feeding jejunostomy (FJ) was performed by an open technique elsewhere to maintain his nutrition.

**Figure 1:**
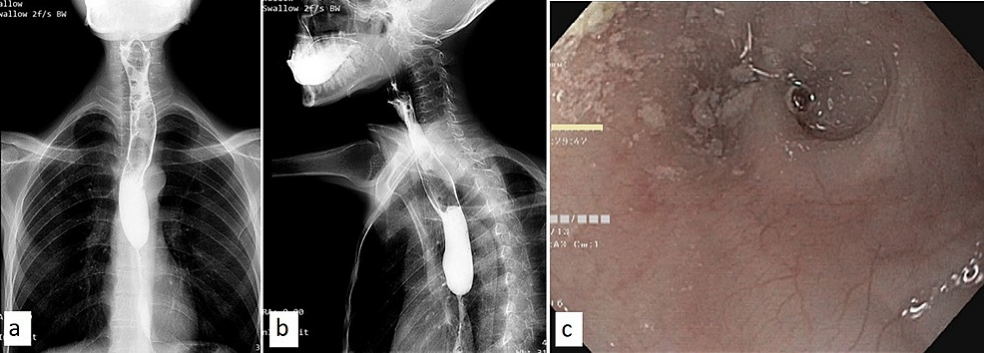
Contrast esophagogram. (a) Anteroposterior and (b) lateral views depicting a long segment esophageal stricture in the lower one-third of the esophagus with contrast hold up proximal to it. (c) Endoscopic view showing stricture in the esophagus.

Subsequently, his oral contrast study and UGI endoscopy suggested a long segment esophageal stricture (approximately 10 cm) in the lower one-third of the esophagus, starting approximately 30 cm from the incisors (Figures 1a-1c). Endoscopic dilatation was endeavored, and the stomach with pylorus was visualized with an ultrathin scope which was essentially normal. However, subsequent attempts to dilate the stricture failed and the decision was taken to restore his enteric continuity with a gastric conduit in view of the long segment stricture, refractory to endoscopic dilatation. Meanwhile, he gained weight after following a high-protein and calorie-rich liquid diet through FJ. As the upper and middle one-third of the esophagus was normal, a decision was made to perform total RILE with intrathoracic anastomosis.

**Figure 2:**
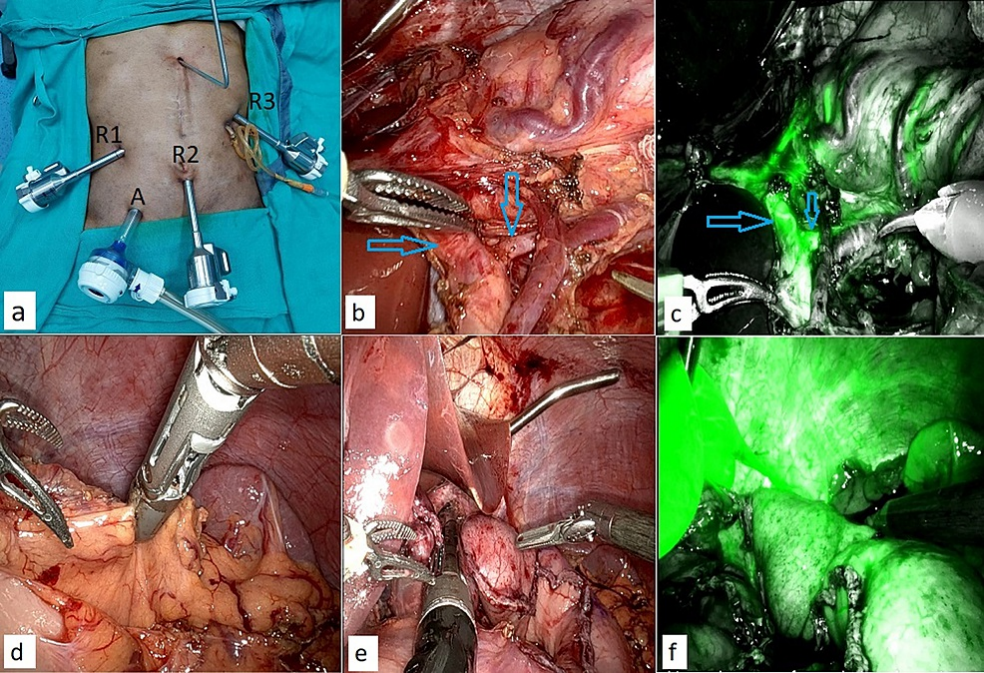
Robotic abdominal phase. (a) Robotic port positions (R1-R3) with one assistant port (A) and feeding jejunostomy in situ. (b) Dissected left gastric vessels (arrow) with accessory left hepatic artery (arrow). (c) Utilization of NIRS mode before dividing the left gastric artery (arrow) and preservation of accessory left hepatic artery (arrow). (d) Division of short gastric vessels with the help of a robotic vessel sealer. (e) Gastric conduit formation with the help of an endostapler. (f) NIRS mode to assess the vascularity of the gastric conduit. NIRS: near-infrared spectroscopy

The robotic abdominal phase was performed with the patient in reverse Trendelenburg and legs split posture. Three robotic and one assistant port were placed with instruments as follows: R1, right pararectus (mid-clavicular line)- Fenestrated bipolar forceps; R2: infra-umbilical camera port; R3, left pararectus (mid-clavicular line)- monopolar curved scissors/vessel sealer; assistant port (A): right pararectus (below and approximately 6 cm away from R1 and R2) (Figure 2a). The Nathanson retractor was used to retract the left lobe of the liver to expose the hiatus. The gastrocolic ligament was divided, keeping the gastroepiploic arcade adjacent to the stomach intact. The left gastric and gastroepiploic vessels were divided with the preservation of the accessory left hepatic artery (Figures 2b-2d). A gastric conduit was formed with the help of Endostapler (Echleon, 60 mm green, Ethicon®, J&J), leaving a thin rim of tissue attached to the specimen for the delivery of the gastric conduit in the chest. Near-infrared spectroscopy (NIRS) mode with indocyanine green (ICG) dye (5 mg) was used to ensure gastric conduit vascularity (Figures 2e, 2f). The abdominal dissection phase was completed after dissecting the hiatus and creating continuity with the mediastinum.

**Figure 3:**
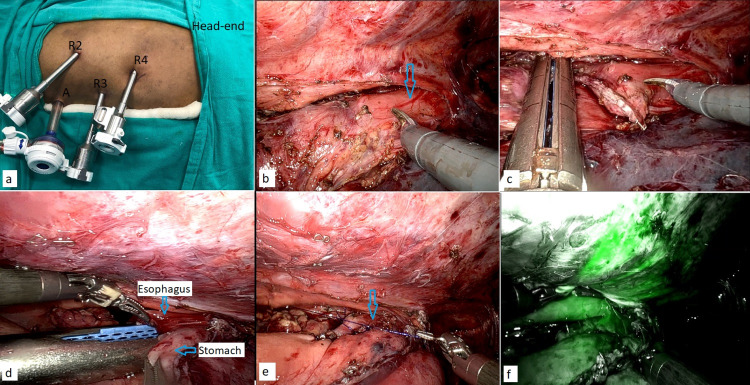
Robotic thoracic phase. (a) Robotic port positions (R2-R4) with one assistant port (A). (b) Demarcation of the diseased and normal esophagus (arrow) after dividing peri-esophageal adhesions. (c) Division of the esophagus with the help of the endostapler. (d) Intrathoracic creation of side-to-side esophagogastric anastomosis with the endostapler. (e) Closure of the common enterotomy site (arrow). (f) NIRS mode to check the vascularity of the anastomotic region. NIRS: near-infrared spectroscopy

After that, the patient was placed in the prone posture with the following robotic port position on the right side of the chest: R2, ninth Intercostal space (ICS) in the posterior axillary line; R3, seventh ICS in the mid-axillary line (camera port); R4, fifth ICS in the posterior axillary line; assistant port (A), a 12 mm port in the ninth ICS mid-axillary line (Figure 3a). On entering the pleural cavity, peri-esophageal adhesions were divided, and the esophagus was mobilized under vision using monopolar curved scissors. The dissection plane was kept adjacent to the esophagus with utmost care not to injure the adjoining structures. The azygous vein was clipped and divided to perform anastomosis in the mid-thoracic esophagus. After complete mobilization of the diseased esophagus, the esophagus was transected above the strictured portion with the help of the endostapler, and the gastric conduit was pulled into the thorax with proper orientation (Figures 3b, 3c). After dividing the left-out small rim of connecting gastric tissue, the specimen was separated. The gastric tube was then placed parallel to the normal esophagus with an anchoring stitch to keep it aligned. The side-to-side esophagogastric anastomosis was made with the help of the endostapler (Echleon, 45mm blue, Ethicon®, J&J) (Figures 3d-3f). A nasogastric tube was placed across the anastomosis, and the common enterotomy was closed with interrupted 4-0 polydioxanone sutures. The specimen was retrieved through the assistant port, a 28 F intercostal drainage (ICD) tube was sited, and the robotic port sites were closed by a skin stapler. The intraoperative blood loss was about 150 mL with a total operative time of seven hours (including 25 minutes of docking time).

**Figure 4:**
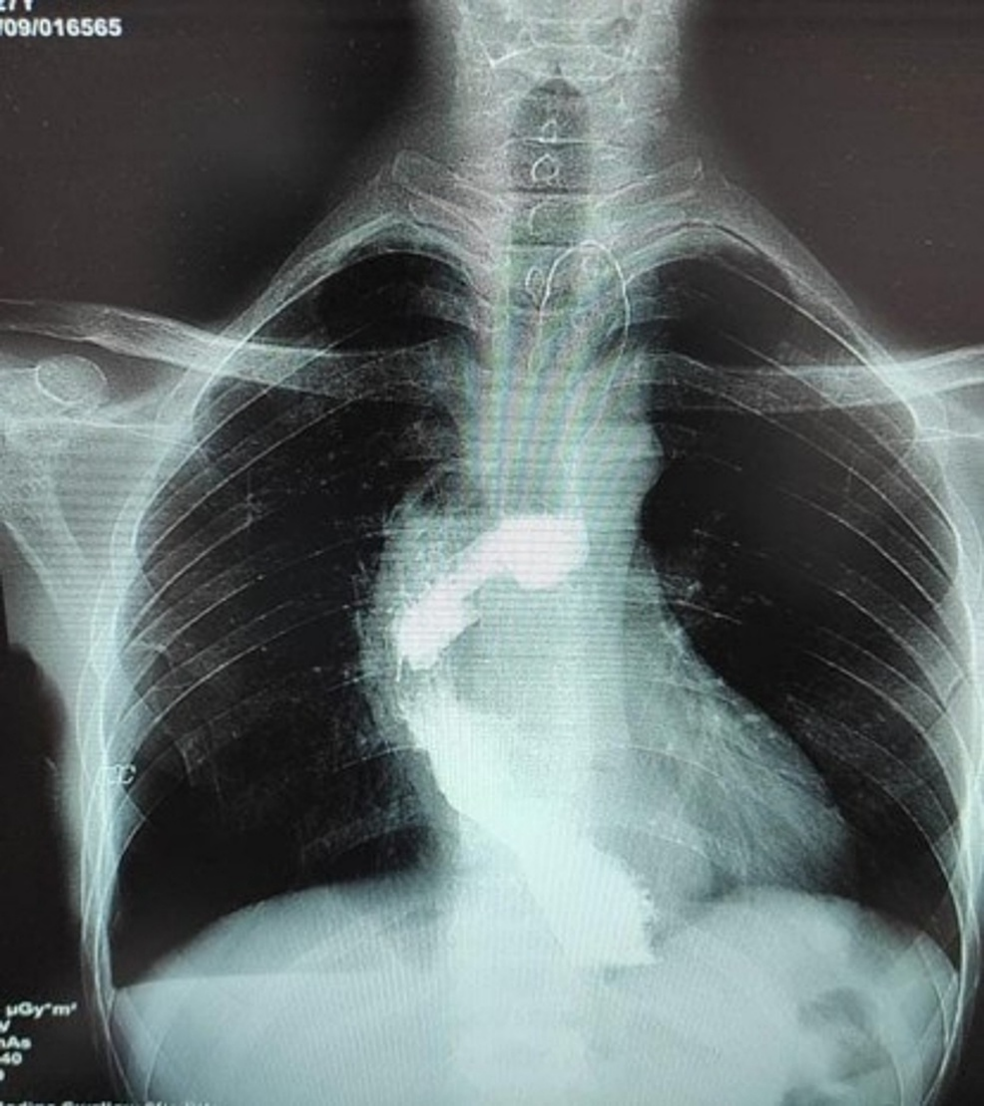
Postoperative oral contrast study. Depicting the free passage of contrast across the anastomotic site with no leakage.

His postoperative course was uneventful, and FJ feeding was commenced on the first postoperative day (POD), which was gradually increased. An oral contrast study was done on the fourth POD, which showed no anastomotic leak, following which he was started on a liquid diet (Figure 4). The ICD tube was removed on the fifth POD, and he was discharged on the seventh POD on a semi-solid diet. At the six-month follow-up, he is accepting a regular diet with weight gain.

## Discussion

CIES is managed mainly by endoscopic dilatation, failure of which mandates surgery. In such a scenario, resection of the esophagus is seldom performed, and a bypass is favored owing to the associated morbidity with esophagectomy [1]. The supporters of esophageal bypass reason that the dissection of dense peri-esophageal adhesions may lead to iatrogenic complications, and the actual incidence of malignancy in the retained esophagus is unlikely to favor the morbid mediastinal dissection [5]. On the other hand, the advocates of esophagectomy claim that although the chances of esophageal cancer are low (lifetime risk of 2-3%), it is crucial as most patients with CIES are young and are at an increased risk over their lifespan [2,6]. Kim et al. reported that the incidence of cicatricial carcinoma (squamous cell carcinoma) was 13%, with the duration between caustic ingestion and diagnosis of cancer ranging from 29 to 46 years [5]. In addition, such patients could not report symptoms of dysphagia early unless it obstructs the conduit or anastomotic site, and endoscopic assessment of the disconnected native esophagus is also not possible. Furthermore, the likelihood of mucocele formation in the left-out esophagus is high when there is a stricture in the lower third of the esophagus with remnant normal mucus-secreting mucosa in the upper and middle third of the thoracic esophagus when a bypass has been performed [2,5]. Good functional and technical outcomes have been reported with esophagectomy done for corrosive esophageal strictures with follow-up of up to 15 years [7].

The stomach is the conduit of choice for replacing the strictured esophagus as it has better outcomes in terms of operative time, anastomotic leak, conduit necrosis, and in-hospital deaths compared to colonic transposition [8]. Further, in the era of advanced laparoscopy and robotic surgery, performing an intrathoracic esophagogastric anastomosis is easier. It avoids the morbidity associated with recurrent laryngeal nerve injury, decreases the risk of aspiration, and has a better-maintained swallowing mechanism compared to neck anastomosis in McKeown esophagectomy [9].

The thoracoscopic approach, either laparoscopic or robotic, in the prone posture for the scarred esophagus is advantageous as it provides a collapsed lung with more space and enhanced visualization for easy peri-esophageal dissection. Further, keeping the dissection plane adjacent to the esophagus under the magnified view prevents injury to important mediastinal structures [4]. In these cases, the conduit length is much more flexible and can be further extended along the fundus as the oncological margin is not of concern. It is superior to its transhiatal counterpart as the critical dissection in the thorax is performed under vision, thereby preventing injury to adjacent structures.

The Ivor-Lewis approach has been utilized in malignancy of the lower third of the esophagus and gastroesophageal junction, and with the recent surge in the use of robotic surgery, intrathoracic anastomosis can be performed effortlessly compared to the open or laparoscopic approach. Open Ivor-Lewis esophagectomy has also been reported for post-corrosive ingestion esophageal perforation and the consequent mediastinitis [6]. Ivor-Lewis esophagectomy has been completed before in the context of CIES only after the development of malignancy in the scarred esophagus [5,10]. We extrapolated a similar technique to manage this benign condition, which has not been previously reported in the literature.

The advantages of RILE in CIES are that the remaining upper and middle third of the normal esophagus is utilized and avoids the complication of mucocele formation. Furthermore, as cervical esophagogastric anastomosis was not performed, this approach avoids a scar in the neck with a preserved swallowing mechanism, leading to earlier oral intake, less frequent aspiration, and lesser consequent pulmonary complications. As the diseased esophagus is resected, it avoids the risk of future malignancy in the remnant esophagus [5]. Moreover, the chances of anastomotic stricture are less in the Ivor-Lewis approach than in McKeown esophagectomy, partly owing to the excellent vascularity of the thoracic esophagus and the conduit not being stretched to reach the neck, enabling the preservation of more vessels in the perigastric region [11]. Some authors have recommended keeping the transection site in Ivor Lewis esophagectomy at or above the level of the azygous vein to avoid the problem of postoperative reflux in these patients [12]. The anastomosis can be easily performed either in a side-to-side manner using endoscopic staplers or end-to-end fashion using Medtronic™ OrVil or robotic staplers. A side-to-side anastomosis is preferred due to the better vascularity on the side, and a wider anastomosis can be created, irrespective of the lumen of the esophagus. We further utilized ICG-NIRS to confirm the gastric conduit’s vascularity and the anastomosis, which helps prevent the disastrous complication of a leak in the mediastinum.

RILE also offers all the advantages of minimal access surgery in the short term such as less pain, earlier ambulation, less wound infection, as well as better cosmesis, body image, and lower risk of incisional hernia in the long term. As CIES is a benign disease, usually occurring in young patients with normal life expectancy, these long-term advantages of RILE become significant.

The limitations of this technique include the selective availability of the robotic platform and the associated learning curve. Though the flexibility offered to the surgeon regarding dissection and reconstruction helps RILE score over other esophagectomy techniques for CIES, further refinement and long-term assessment of this approach need to be ascertained.

## Conclusions

RILE with gastric pull-up is a feasible option in selected cases of CIES with stricture in the lower part of the esophagus, especially in young patients. In addition to the ease of performing the procedure, it helps to avoid the chances of mucocele development and sequelae of cervical neck anastomosis.
